# Patient Blood Management improves outcome in oncologic surgery

**DOI:** 10.1186/s12957-018-1456-9

**Published:** 2018-08-07

**Authors:** Vivienne Keding, Kai Zacharowski, Wolf O. Bechstein, Patrick Meybohm, Andreas A. Schnitzbauer

**Affiliations:** 1 Clinic for General and Visceral Surgery, University Hospital Frankfurt, Goethe University Frankfurt/Main, Theodor-Stern-Kai 7, 60590 Frankfurt/Main, Germany; 2 Department of Anesthesiology, Intensive Care Medicine, and Pain Therapy, University Hospital Frankfurt, Goethe University Frankfurt, Frankfurt/Main, Germany

**Keywords:** Patient Blood Management, Oncologic surgery, Transfusion

## Abstract

**Background:**

Patient Blood Management (PBM) is a systematic quality improving clinical model to reduce anemia and avoid transfusions in all kinds of clinical settings. Here, we investigated the potential of PBM in oncologic surgery and hypothesized that PBM improves 2-year overall survival (OS).

**Methods:**

Retrospective analysis of patients 2 years before and after PBM implementation. The primary endpoint was OS at 2 years after surgery. We identified a sample size of 824 to detect a 10% improvement in survival in the PBM group.

**Results:**

The analysis comprised of 836 patients that underwent oncologic surgery, 389 before and 447 after PBM, was implemented. Patients in the PBM+ presented significantly more frequent with normal hemoglobin values before surgery than PBM− (56.6 vs. 35.7%; *p* < 0.001). The number of transfusions was significantly reduced from 5.5 ± 11.1 to 3.0 ± 6.9 units/patient (*p* < 0.001); moreover, the percentage of patients being transfused during the clinic stay was significantly reduced from 62.4 to 40.9% (*p* < 0.001). Two-year OS was significantly better in the PBM+ and increased from 67.0 to 80.1% (*p* = 0.001). A normal hemoglobin value (> 12 g/dl in female and > 13 g/dl in male) before surgery (HR 0.43, 95% CI 0.29–0.65, *p* < 0.001) was the only independent predictive factor positively affecting survival.

**Conclusions:**

PBM is a quality improvement tool that is associated with better mid-term surgical oncologic outcome. The root cause for improvement is the increase of patients entering surgery with normal hemoglobin values.

**Electronic supplementary material:**

The online version of this article (10.1186/s12957-018-1456-9) contains supplementary material, which is available to authorized users.

## Background

The discussion of whether a liberal or restrictive transfusion regimen adversely or positively affects the patient outcome is long lasting in medicine. In 1999, Hébert et al. published one of the first randomized controlled trials showing that a restrictive strategy of red-cell transfusion is at least as effective as a liberal transfusion strategy in critically ill patients [1]. Other authors confirmed these findings for different indications, e.g., septic shock and large cohorts detected the application of already 1 unit of blood as an independent risk factor for increased morbidity and mortality [2, 3]. Recently, a large national initiative was launched in Germany: the so-called Patient Blood Management project to increase patient safety. In a first prospective analysis in surgical patients, it was shown that more careful handling of red blood cells with adjusted and strict triggers for transfusion did not increase morbidity and mortality. Moreover, an algorithmic approach to minimize anemia before surgery in patients scheduled for elective surgery was established. All these measures together led to a significant reduction in the application of blood products, resulting in a relevant potential for economization [4–6]. Specifically, Meybohm et al. and other authors showed that the use of 1 unit of blood during general surgical procedures already led to an increase in morbidity and mortality of patients [7].

Besides the clinical and economic evidence of PBM, transfusions may also have immunologic effects that increase morbidity and mortality, e.g., an enhanced recurrence rate after tumor resection [8]. Dixon et al. named the RBC transfusion rate as a neglected potential quality parameter of outcome in oncologic surgery [9]. Nevertheless, there is no clinical evidence that a structured program of PBM may lead to an improved long-term outcome in oncologic surgery [10]. Therefore, we analyzed all patients undergoing elective surgery for oncologic indications. We hypothesized that there is a consistent improvement in 2 years overall survival of at least 10% after PBM implementation.

## Methods

### Patient selection, the period of evaluation and data-collection, endpoints

All consecutive inpatients (aged ≥ 18 years) undergoing abdominal oncologic surgery were included in the analysis, 24 months before and after implementation of PBM at University Hospital Frankfurt [4]. The cutoff date for the implementation of PBM was on July 1, 2013.

ICD-10- and OPS-codes had to refer to malignant disease. If a patient had multiple hospital admissions during the study period, only the first hospital stay was included to avoid overlap. Surgical procedures were classified according to the German surgery and procedure classification, based on the International Classification of Procedures in Medicine.

Data collected were age, gender, indications for resection (hepatobiliary, colorectal liver metastases, pancreatic, gastric, intestinal, esophagus, primary other and metastasis other), history of concomitant disease in accordance with ICD10 coding (cardiovascular I00-I99, pulmonic J00-J99, endocrine E00-E35, gastrointestinal K00-K93, renal N00-N29, hematologic D50-D90, malignant other C00-C97, infection A00-B99), hemoglobin prior to and post surgery, percentage of patients with a normal hemoglobin value prior to and post surgery, number of RBC units transfused until hospital discharge, percentage of patients receiving at least 1 RBC unit, the complication rate in accordance with the classification of Dindo and Clavien as well as 30-day, 90-day, and overall survival rates. The ethics review board (Ethikkommission des Fachbereichs Medizin) granted permission for analysis (number 218/17, dated July 17, 2017).

The primary endpoint was 2-year overall survival. Secondary endpoints were 30-day and 90-day survival, the percentage of patients with anemia, number of RBC units transfused, the percentage of patients with RBC transfusion, and complication rates following the classification of Dindo and Clavien [11].

#### Intervention––Patient Blood Management

Patient Blood Management is a clinical quality program. The implementation of a structured Patient Blood Management included six bundles. As a first bundle, dedicated project management with involvement of crucial PBM stakeholders was founded. Education included undergraduate and post graduate teaching as well as the establishment of local standards and protocols. Moreover, bundle 2 consisted of specific diagnosis and treatment of anemia. Bundle 3 focused on management of coagulopathy during surgery. Bundles 4 and 5 mainly yielded at the reduction of diagnostic-associated blood loss and reduction of surgery associated blood loss. Finally, outcome measures were defined in bundle 6 including the endpoints targeted in this study. Exact information can be obtained in the English version of https://www.patientbloodmanagement.de/en/pbm-bundles/ and was published by the group elsewhere [12]. The PBM program focused on preoperative optimization of hemoglobin levels, blood-sparing techniques, standardization of transfusion practice, and regular education sessions. Compliance with guideline-based transfusion triggers was supervised by electronic-based checklists, in which the indication of each RBC transfusion had to be documented in the patient’s record.

In brief, if a patient has a hemoglobin value of < 12 (f) or < 13 (m) g/dl and the transfusion probability is > 10%, iron status is measured. In case iron deficiency as the leading course for anemia was present, iron i.v. was supplemented. Intraoperative and postoperative thresholds for transfusion were adjusted to hemoglobin < 6 g/dl in the absence of other triggers like shock or dyspnea and 6 to 8 g/dl in case a patient has specific risk factors or signs for hypoxia. These bundles applied for the indication of every single transfused RBC. The exact algorithm is displayed in the Additional file 1: Figures S1 and S2.

### Data management and statistical analysis

Data were extracted from the electronic patient charts. For survival data, the University Cancer Center database was used to identify patient follow-up and status. In case a patient was lost to follow up, the date of the last known and documented status was used. To estimate the power, sample size calculations for the validity of the findings were made based on 2-year overall survival data. We estimated that the average 2-year overall survival probability was 70% for all surgical oncologic procedures. Considering a 10% benefit in patients with PBM, a two-sided alpha-value of 0.05, and a beta-value of 0.20 reflecting the power of 80%, overall 824 patients were necessary for analysis.

Differences in demographics were detected using paired *t* tests, Fisher’s exact test, and the Pearson *Χ*^2^ test. Demographic data are given as means with standard deviation or distribution in percentage between the groups. Kaplan-Meier-estimations were used to detect differences in 2-year, 30-day, and 90-day overall survival between the groups. Patients dying within 2 years after surgery were censored for death; patients lost to follow up were censored alive on the day of the last follow-up. Univariate and multivariate analysis were performed using COX regression analysis with stepwise backward elimination. *P* values < 0.05 were defined as statistically significant. Data were analyzed with SPSS Version 23.0 (IBM, New York, USA).

## Results

### Patients and baseline demographics

Between July 1, 2011, and July 1, 2015, a total of 7041 cases were treated in the Clinic for Abdominal and Visceral Surgery at University Hospital Frankfurt. A total of 6662 surgeries were coded and performed. Of those, 836 patients were treated for malignant diagnosis and underwent oncological surgery with a curative approach. Of the 836 patients included, 389 were included in the pre-PBM cohort (PBM−) and 447 in the PBM cohort (PBM+). Indications are displayed in Table 1 and were equally distributed between groups.

**Table 1:** Demographic data, indications, concomitant disease, pre-surgical anemia, and numbers of RBC transfusions

	Cumulative *N* = 836		PBM− *N* = 389		PBM+ *N* = 447		*p* value
Age (years)			64.8 ± 13.6		66.9 ± 12.4		0.019
Gender (m/f) (%)	508 (63.1%)	328 (39.2%)	216 (55.5%	173 (44.5%)	292 (65.3%)	155 (34.6%)	0.004
Indication							
Hepatobiliary	212 (25.7%)		92 (23.7%)		120 (26.8%)		0.06
Pancreatic	80 (9.5%)		48 (12.3%)		32 (7.1%)		0.09
CRLM	273 (32.6%)		136 (35.0%)		137 (30.6%)		1.00
Upper GI	99 (11.8%)		54 (13.9%)		45 (10.0%)		0.42
Intestinal	112 (13.4%)		38 (9.8%)		74 (16.6%)		0.001
Primary other	22 (2.6%)		14 (3.6%)		8 (1.8%)		0.29
Metastases other	38 (4.5%)		7 (1.8%)		31 (6.8%)		< 0.001
Concomitant disease							
Cardiovascular	437 (52.6%)		212 (54.5%)		225 (51.0%)		0.229
Pulmonic	86 (10.1%)		48 (12.3%)		38 (8.3%)		0.068
Endocrine	232 (27.6%)		97 (25.1%)		135 (29.8%)		0.090
Gastrointestinal	319 (38.1%)		178 (45.8%)		141 (31.5%)		< 0.001
Renal	71 (8.4%)		24 (6.1%)		47 (10.3%)		0.034
Hematologic	50 (6.0%)		27 (7.2%)		23 (5.0%)		0.246
Infection	84 (10.3%)		38 (9.7%)		46 (10.7%)		0.802
Hemoglobin prior to surgery (g/dl)			11.9 ± 2.2		12.5 ± 1.9		< 0.001
Hemoglobin before surgery normal			139 (35.7%)		253 (56.6%)		< 0.001
RBCs transfused per patient			5.5 ± 11.1		3.0 ± 6.9		< 0.001
Patients receiving at least 1 RBC			242 (62.4%)		180 (40.9%)		< 0.001
Complications DC > 3a (major)	142 (16.9%)		70 (17.9%)		72 (16.0%)		0.463

*PBM* Patient Blood Management, *CRLM* colorectal liver metastases, *GI* gastrointestinal, *RBC* red blood cells, *DC* Dindo-Clavien

### Overall survival of patients

**Fig. 1:**
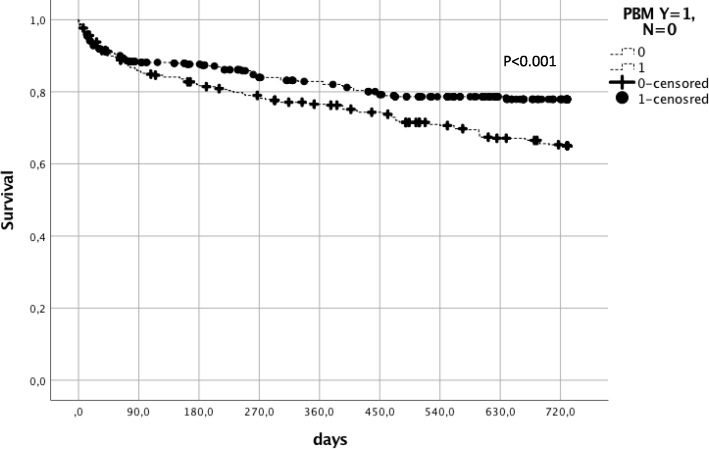
Two-year overall survival comparing patients with and without Patient Blood Management

**Fig. 2:**
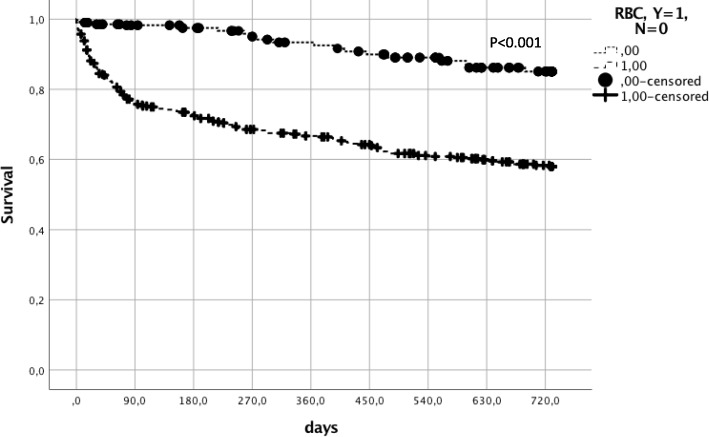
Two-year overall survival comparing patients with and without transfusion. PBM Patient Blood Management, Y yes, N no, RBC red blood cells

Mean overall follow-up was 43.6 ± 1.5 months in PBM− and 34.1 ± 0.8 months in PBM+ associated with significant differences in overall survival of 61.6 and 78.6% (*p* < 0.001). Two-year overall survival was 73.9% in all patients, 66.8% in PBM−, and 80.1% in PBM+ (*p* = 0.001). In total, 129 patients died in PBM− and 89 patients in PBM+ within 2 years after surgery (Fig. 1). Notably, 30-day and 90-day mortality rates were not different between the investigated groups (92.8 vs. 91.9%; *p* = 0.595 and 85.7 vs. 87.7%; *p* = 0.444). Patients that were transfused had a significantly better 2-year overall survival (87.5 vs. 61.0%, *p* < 0.001). The trend was consistent in both the PBM-era (90.7 vs. 64.7%, *p* < 0.001) and in the non-PBM-era (81.6 vs. 58.2%, *p* < 0.001) (Fig. 2). However, there was a large transfusion sparing effect of more than 20% after the PBM program was introduced.

### Secondary endpoints

There was a definite trend towards higher hemoglobin levels in the PBM+ group before surgery, which is an effect of the structured quality program. The number of patients with normal hemoglobin was significantly higher in PBM+ (56.6 vs. 35.7%, *p* < 0.001). The number of transfused RBCs/patient was significantly lower in the PBM+ group (5.5 ± 11.1 vs. 3.0 ± 6.9; *p* < 0.001), and the number of transfused patients was also significantly lower (62.4 to 40.9%; *p* < 0.001). Complications (Dindo-Clavien>IIIa) were not different between the groups.

### Age, gastrointestinal concomitant disease, normal hemoglobin before surgery, complications, and the number of transfused RBCs are independent predictors for 2-year overall survival

Univariate analysis identified 11 factors that were associated with outcome. Factors being significant were included in a multivariate analysis, which revealed increasing age (HR 1.02, 95% CI 1.00–1.04, *p* = 0.008), the presence of gastrointestinal concomitant disease (HR 1.86, 95% CI 1.26–2.76, *p* = 0.002), the number of transfusions/patient (HR 1.03, 95% CI 1.00–1.05, *p* = 0.023), and the presence of major surgical complications (HR 7.52, 95% CI 4.50–12.57, *p* < 0.001) as independent risk factors for death; a normal hemoglobin value before surgery (HR 0.43, 95% CI 0.29–0.65, *p* < 0.001) was associated with improved overall survival (Table 2). A ROC analysis revealed an AUC-ROC of 0.595 for age and 0.729 for the number of transfused RBC units. For the number of units transfused, a cutoff of 1 unit of transfusion was identified as a threshold for impaired survival reflecting a sensitivity of 75% and a specificity of 61%.

**Table 2:** COX regression analysis of univariate and multivariate factors influencing 2-year overall survival

	Univariate analysis				Multivariate analysis			
Parameter	HR	95% CI lower	95% CI upper	*p* value	HR	95% CI lower	95% CI upper	*p* value
Age (years)	1.03	1.015	1.039	< 0.001	1.02	1.00	1.04	*0.02*
Gender	0.94	0.71	1.25	0.68				
Transfusion of RBC	3.53	2.55	4.87	< 0.001	0.77	0.39	1.54	0.46
Endocrine concomitant disease	0.98	0.71	1.33	0.87				
GI concomitant disease	2.07	1.56	2.73	< 0.001	1.68	1.1	2.57	*0.016*
Hematologic concomitant disease	1.21	0.67	2.17	0.53				
Infection concomitant disease	0.95	0.60	1.49	0.81				
Cardiovascular concomitant disease	0.94	0.71	1.25	0.68				
Renal concomitant disease	1.61	1.01	2.56	0.04	1.32	0.68	2.57	0.42
Pulmonic concomitant disease	0.80	0.49	1.30	0.37				
Malignancy concomitant disease	1.63	1.24	2.16	0.001	0.88	0.52	1.48	0.62
PBM yes	0.66	0.50	0.90	0.006	0.89	0.48	1.65	0.71
Hemoglobin prior surgery	0.83	0.74	0.93	0.001	0.98	0.80	1.19	0.80
Hemoglobin post surgery	0.66	0.57	0.76	< 0.001	0.94	0.80	1.12	0.50
Hemoglobin prior surgery normal	0.49	0.32	0.75	0.001	0.48	0.31	0.74	*0.001*
Hb post surgery normal	1.40	0.70	2.78	0.34				
Complications > DC IIIA without V	8.90	6.69	11.85	< 0.001	12.39	7.88	19.48	**<** *0.001*
Total number of RBCs	1.1	1.04	1.06	< 0.001	1.02	0.99	1.04	0.21

*HR* hazard ratio, *CI* confidence interval, *GI* gastrointestinal, *PBM* Patient Blood Management

### Subgroup analysis

#### Patients with minor complications may profit most from PBM

In total, 705 (84.3%) patients experienced minor complications (<Dindo-Clavien IIIb), (384 in PBM+ and 321 in PBM−). Two hundred ninety-seven patients in the minor-complication group (42%) were transfused, whereas 408 (58%) did not receive an RBC. Patients with minor complications had better oncologic outcome in the PBM+ (88.3 vs. 75.7%; *p* < 0.001) and without being transfused (88.7 vs. 74.1%; *p* < 0.001).

Notably, 142 patients (16.9%) experienced major complications (>Dindo-Clavien IIIa), of which 134 (94%) received at least one transfusion. Major complications were present in 70 patients in PBM− and 73 in PBM+. When patients experienced major complications, overall survival decreased, independently from RBC transfusion (33.3 vs. 32.1%; *p* = 0.669) or PBM−/PBM+ (37.0 vs. 27.1%; *p* = 0.850).

#### PBM improved 2-year overall survival for the majority of indications

Regarding indications, there was an improvement in survival after 2 years for mostly all indications after PBM (*p* = 0.001). Liver diseases without colorectal liver metastases (CRLM) improved from 63.0 to 69.2% and CRLM from 71.3 to 85.4%. Overall survival for pancreatic malignancies improved from 56.3 to 68.8%, for upper-GI-indications from 71.7 to 84.8% for gastric cancer, and from 37.5 to 58.3% for esophageal cancer. Intestinal and colorectal cancer indications improved from 68.4 to 91.9%. Results for other cancers (78.6 to 87.5%) and other metastases (71.4 to 83.9%) were also improved.

## Discussion

In this retrospective analysis of > 800 patients undergoing oncologic surgery, the implementation of a structured PBM program led to a significant reduction in RBC transfusion requirements. This reduced need for transfusions was associated with a significantly improved 2-year survival by 15%, while short-term surgical outcomes were not affected. Transfusion thus may be an early determinant on late outcome. The number of patients receiving RBC could be significantly reduced (20%) after PBM implementation, and the number of patients starting with normal hemoglobin was significantly higher in the PBM cohort, which reduced the risk for 2-year mortality by 50%. In patients with minor complications, the benefit of PBM could also be proven. Uncritical transfusion practice in these patients, however, was associated with adverse outcome.

In contrast, patients with significant complications had a dramatically decreased survival rate of about 30–35%. Almost all patients received at least one transfusion. In most patients, transfusion was necessary in case of a life-threatening condition, e.g., massive bleeding. Not surprisingly, major complications were associated with adverse outcome, while PBM and transfusion practice had no additional impact on survival.

In the literature, transfusions were associated with increased morbidity and mortality. Sutton et al. described an adverse outcome in pancreatic cancer (overall survival: 14 vs. 21 months) [13]. Other authors confirmed this for various indications: Martin et al. (CRLM: odds ratio (OR) 4.18, 95% CI 2.18–8.02) and mortality (OR 14.5, 95% CI 3.08–67.8) [14], Schiergens et al. (reduced recurrence-free survival (32 vs. 72 months in CRLM) [15], and Reim et al. (gastric cancer: hazard ratio (HR) 1.31, 95% CI 1.01–1.69) [16]. However, these findings were based on dichotomization of data and not on the introduction of a structured program to reduce transfusions, which is the crucial difference to our work. Our investigation concentrated on a real-life scenario of an era with Patient Blood Management that aims at minimization of perioperative transfusions and compares it with an era where this program has not been present in our clinic. Data in the non-transfusion group thus may even be better. However, the systematic introduction of PBM (still including patients with transfusions, but reduced by 20%) naturally reached similar results than cohorts with no transfusion at all, by avoiding transfusions in patients that have an increased risk instead of benefit from sometimes––necessary blood supplementation in daily clinical practice.

This is reflected by beneficial survival data for individual indications, which were also in good agreement with data in the literature: Jarnagin et al. (intrahepatic and perihilar cholangiocarcinoma, 63 and 69% [17]; Zaydfudim et al. (hepatocellular carcinoma, 6059 [18]; Schiergens et al. and Margonis et al. (CRLM 68% with transfusion and 82% without transfusion) [15, 19]; esophageal cancer (40% with transfusion vs. 60% without transfusion) [20]; gastric cancer (82 vs. 60%) [16]; and pancreatic cancer (50 to 60% vs. 20%) [13, 21]. Last not least, Mörner et al. showed that anemia and transfusions were associated with adverse outcome [22]. Moreover, Mörner and also Wilson stress that pre-surgical normal hemoglobin is an essential reducer for the risk of death after surgery, which is also in perfect agreement with our data [22, 23].

Nonetheless, there are some limitations to the study, like its retrospective nature in however without selection for indications or concomitant disease. Positively, the sample size calculation aimed at analysis of 845 patients to be able to detect a significant difference of at least 10% in overall survival. Therefore, this retrospective single-center cohort is a good indicator of the potential of PBM. Undoubtedly, the awareness for a responsive transfusion practice has sustainably been established. Where other studies describe a benefit of restricted transfusion regimens more as a coincidental finding, this is the first report in which a complete system in a clinic was changed, and results of this change by otherwise stable conditions can be shown.

From a clinical perspective, our policy is that every surgical procedure has to be at least assisted by a board-certified surgeon and that steps of every operation have to be assisted whenever possible. Data from various analysis of the American NSQIP database show that outcomes are not influenced by resident involvement [24]. Moreover, every patient requires presentation in a multidisciplinary tumor board. As we are one of two German University Clinics that are certified for every abdominal tumor by the German Cancer Society, our pre-therapeutic discussion rate in multidisciplinary boards is close to 100%. Post procedural multidisciplinary boards and annual audits by the German Cancer Society for recertification do not show personnel or oncologic indication-specific variations.

Unfortunately, the mechanisms that lead to the improved outcome in the oncologic surgical cohort after PBM remain unclear and speculative. In general, the assumed immunologic effects of long-term improvement are unclear and not backed-up by refined translational clinical trials of the mechanisms leading to tumor growth, tethering and dissemination caused by transfusion, or a complicated clinical course. Goubran et al. therefore called for substantial retrospective data analysis and well-designed prospective translational trials to clarify the yet unclear mechanisms of transfusion-tumor interaction on an immunologic level [25].

## Conclusion

In conclusion, this retrospective analysis shows that a complex PBM program focusing on normal hemoglobin before surgery, multimodal blood-sparing techniques, and a rationale transfusion regimen improve outcome after oncologic surgery. Presumed that evidence will be further increased, PBM may be a future key element of long-term patient safety and outcome in a multidisciplinary setting of oncologic surgery.

## Additional file

Additional file 1: **Figure S1.** Algorithm to detect and treat preoperative iron deficiency with/without anemia currently in use at the University Hospital Frankfurt. **Figure S2.** Checklist of transfusions triggers currently in use at the University Hospital Frankfurt. (PPTX 791 kb)
