# Supplementary material for: Patient Blood Management improves outcome in oncologic surgery
Source: World J Surg Oncol. 2018 Aug 7;16:159. doi: 10.1186/s12957-018-1456-9 (PMC6081799; doi:10.1186/s12957-018-1456-9)
Supplement: Supplementary file 1 — Figure S1. Algorithm to detect and treat preoperative iron deficiency with/without anemia currently in use at the University Hospital Frankfurt. Figure S2. Checklist of transfusions triggers currently in use at the University Hospital Frankfurt. (PPTX 791 kb) [file 12957_2018_1456_MOESM1_ESM.pptx]

## Slide 1
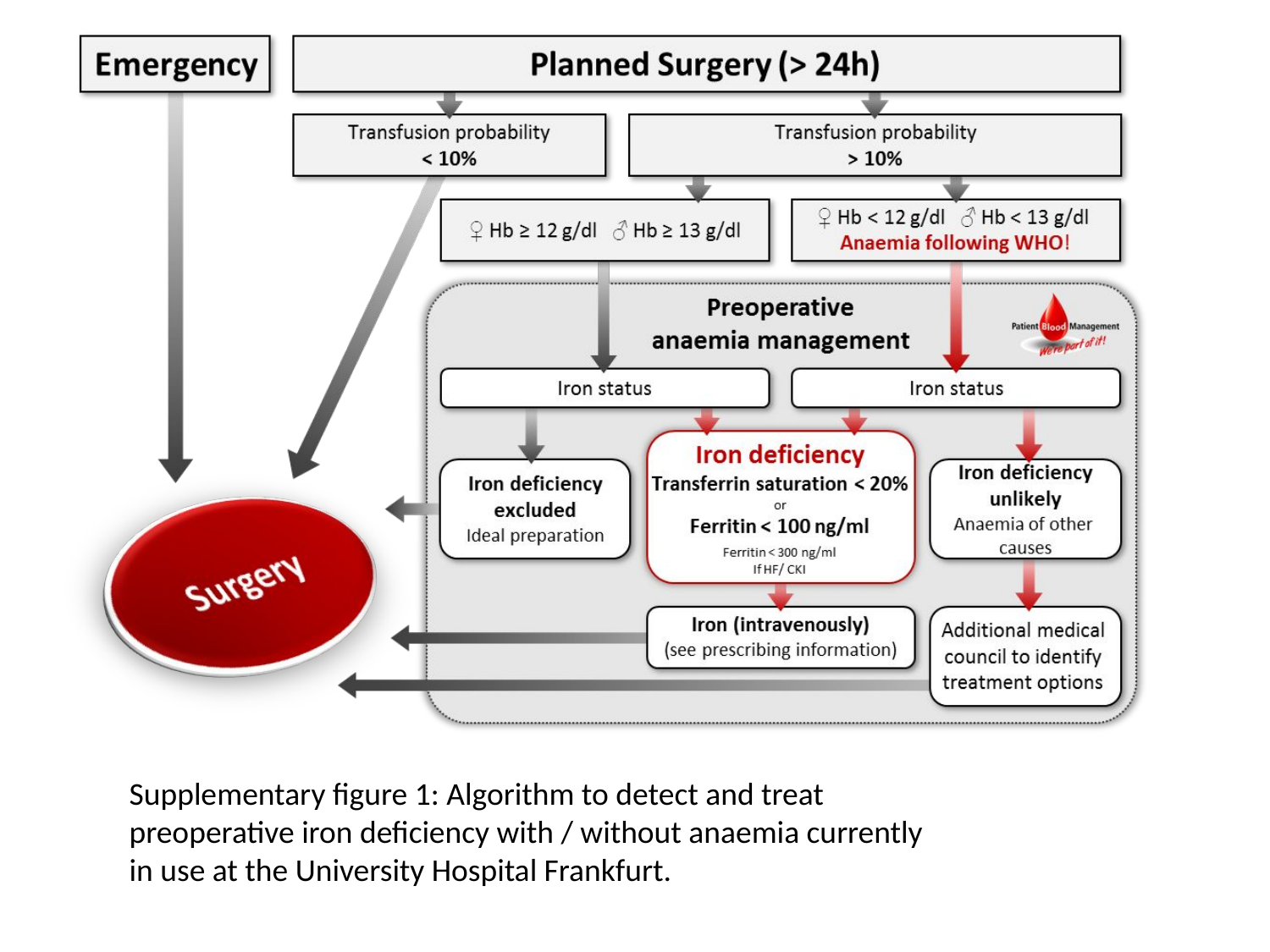

Supplementary figure 1: Algorithm to detect and treat preoperative iron deficiency with / without anaemia currently in use at the University Hospital Frankfurt.

## Slide 2
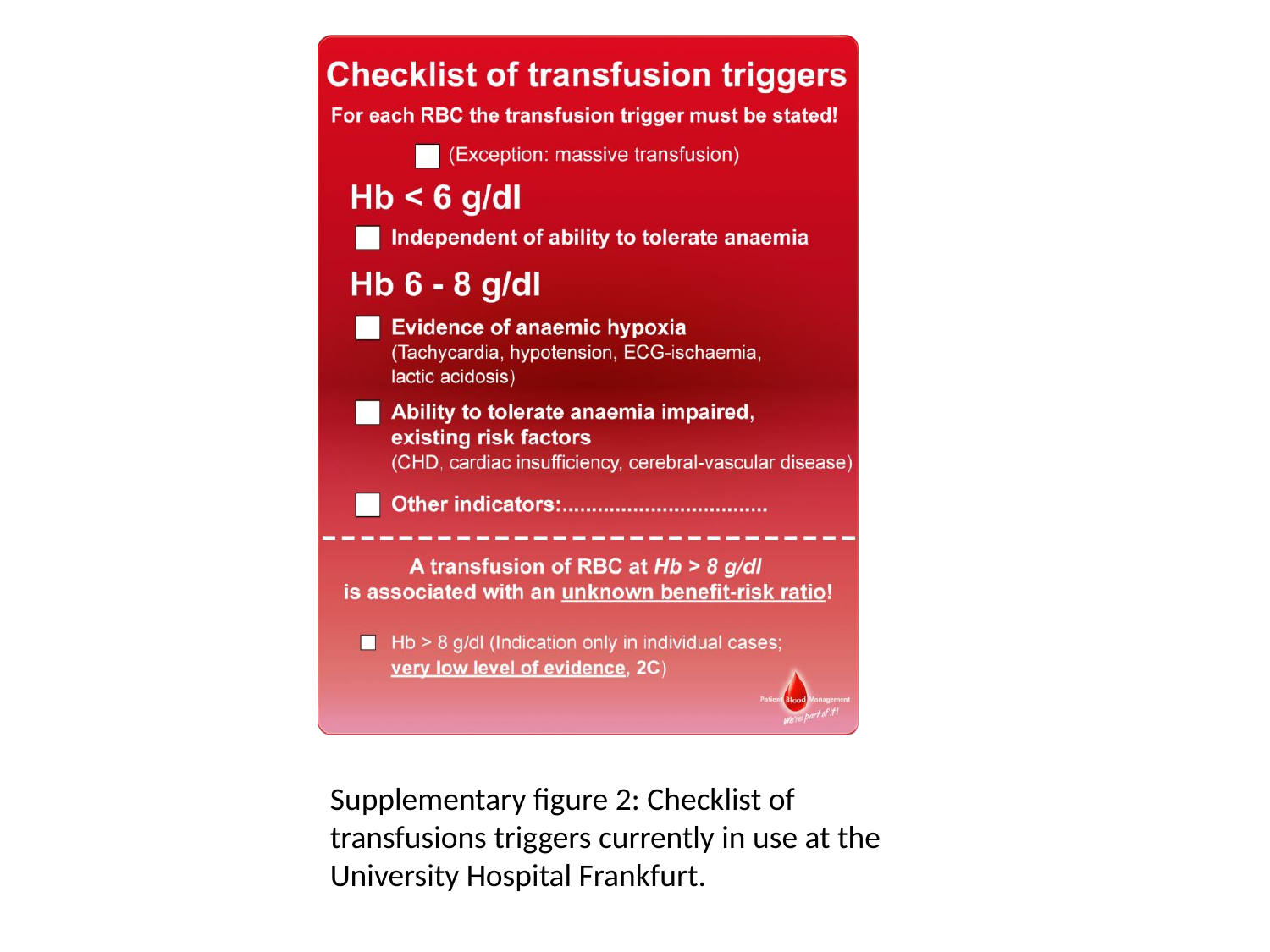

Supplementary figure 2: Checklist of transfusions triggers currently in use at the University Hospital Frankfurt.
